# Motivated Cue-Integration and Emotion Regulation: Awareness of the Association Between Interoceptive and Exteroceptive Embodied Cues and Personal Need Creates an Emotion Goal

**DOI:** 10.3389/fpsyg.2020.01630

**Published:** 2020-07-10

**Authors:** Idit Shalev

**Affiliations:** Laboratory for Embodiment and Self-Regulation, Department of Psychology, Ariel University, Ariel, Israel

**Keywords:** motivated cue-integration, emotion regulation, emotional clarity, awareness of sensation techniques, self-regulation, multisensory, interoception

## Abstract

Research on emotion suggests that individuals regulate their emotions to attain hedonic or instrumental goals. However, little is known of emotion regulation under low emotional clarity. The theory of motivated cue integration (MCI) suggests that emotion regulation under low emotional clarity should be understood as dissociation between a high-level individual hierarchical system of goals and low level interoceptive and exteroceptive embodied cues. MCI conceptualizes low emotional clarity as the product of low access to signals of emotion that result in prediction error associated with mismatch between the current bodily state and the predicted state. This deficit in emotional processing could be understood as a problem of means substitution, suggesting that use of alternative multisensory data may facilitate situational evaluation. Based on this reasoning, a new perspective on emotion regulation under low emotional clarity is presented, according to which interchangeable attention to multisensory data associated with words, associations, and images may help in cue integration, enabling the creation of a link between concrete bodily cues, abstract mental representation, and a more accurate prediction. Based on the idea that emotional episodes are conceptualized as special types of goal-directed action episodes, this process will lead to the creation of broader integrative meaning, results in the development of emotion goal.

## The Problem Of Low Emotional Clarity

Emotion regulation is an essential aspect of mental health and refers to processes that amplify, attenuate, or maintain the strength of emotional reactions (Gross, 1998; Davidson et al., 2000) such that individuals are capable of controlling their behavior (see Linehan, 1993; Melnick and Hinshaw, 2000) or accepting and valuing emotional responses (Zajonc, 1980; Schwarz, 1990; Linehan, 1993; Cole et al., 1994). To date, ample research on emotion regulation has mainly been focused on top-down strategies aimed to change current emotions into desired emotions (see Gross, 2015). For example, the instrumental approach of emotion regulation suggests that individual emotions are motivated by hedonic or instrumental benefits to promote goal pursuit (e.g., Tamir and Ford, 2009, 2012; Tamir and Bigman, 2018). Research sheds light on the differences between implicit and explicit regulation of emotion, indicating the extent of effort under explicit regulation of emotion (Gyurak et al., 2011; Koole and Rothermund, 2011). However, little is known about the transition mechanisms between low and high emotional clarity to establish an emotional goal and better regulatory control. There is evidence of the differentiation between six levels of regulatory control (Lane et al., 2000; Smith and Lane, 2015), indicating that the lowest level of regulatory control is the automatic level of somatic and visceral reflexes associated with body state‐ and behavioral-regulation (Longhurst, 2011; Strominger et al., 2012). In fact, only the sixth and highest level of regulation is associated with voluntary emotion regulation (Smith and Lane, 2015), with its activation depending on having an emotion goal and strategic goal-directed attempts to suppress or reappraise emotional responses. This level is associated with top-down voluntary control over cognition, attention, and behavior, which activates regions such as dorsolateral prefrontal cortex (DLPFC), the dorsomedial prefrontal cortex (DMPFC), the ventrolateral prefrontal cortex (VLPFC) and parts of the dorsal anterior cingulate cortex (dACC) and the posterior parietal cortex (Gross, 1998; Ochsner and Gross, 2005).

Emotional clarity is defined as the extent to which people unambiguously identify, label, and characterize their own emotions. A majority of theories consider awareness and clarity of emotions as the building blocks of emotion regulation (e.g., Salovey et al., 1995; Gratz and Roemer, 2004, 2008; Boden and Thompson, 2015). For example, the “process model” (Gross, 1998) suggests that appraisal of a situation involves assessment of cues associated with emotional response against top-down prevailing factors (e.g., goals, social, cultural and familial influences, personality, etc.,). Accordingly, there is evidence that individuals high in clarity of emotion have meta-emotional knowledge that facilitates efficient and effective selection and implementation of emotion regulation strategies which have the greatest likelihood of achieving emotion regulation goals (Barrett et al., 2001; Tamir et al., 2008; Gross and Jazaieri, 2014). By contrast, when clarity of emotion is low, people tend to not regulate emotions or to fail in effective selection and implementation of emotion regulation strategies (Boden et al., 2013). Research demonstrates that emotional clarity deficits are associated with symptoms of depression, social anxiety, borderline personality, binge eating, and alcohol use, indicating that emotional clarity deficits may be viewed as a transdiagnostic phenomenon with divergent pathways causing problems in controlling emotions ranging from condition to condition (Vine and Aldao, 2014). These deficits come into play especially in alexithymia (Murphy et al., 2018), viewed as a continuum which manifests as difficulty in identifying feelings and in distinguishing them from bodily sensations of emotional arousal, difficulty in describing one’s own feelings, and an externally oriented cognitive style, i.e., a focusing of one’s attention externally with little introspection or insight into one’s own feelings (Nemiah et al., 1976). Following this view, there is evidence that lower vagally mediated heart rate variability (vmHRV) resting is linked to enhanced emotional regulatory difficulties. Particularly, deficient in emotional clarity and impulse regulation, indicating that emotional regulation and autonomic regulation share neuronal networks within the brain (Williams et al., 2015). The influence of poor emotional clarity was also identified in psoriasis patients with high psychological distress. They were lower in emotional clarity than control and displayed less performance discrepancy under low vs. high cognitive load conditions (Panasiti et al., 2019).

Motivated cue integration (MCI) theory (Shalev, 2015, 2018, 2019) provides a perspective on the potential transformation of low emotional clarity into the creation of emotion goals. In what follows, I will first present the theory of MCI. I will then apply the principles of the theory on the problem of low emotional clarity. Based on the principles of the theory, I will finally propose therapeutic strategies to increase emotional clarity.

## Theory of Motivated Cue Integration

The theory of MCI (Shalev, 2015, 2018, 2019) attests that individuals construe meaning by integrating high-level control processes and low-level cues, such that, on the one hand, active top-down goals influence attention to interoceptive and exteroceptive signals. On the other hand, the perceiver’s likelihood of drawing a specific inference is based on her unique system of goals, suggesting that active goals increase the accessibility of specific interoceptive and exteroceptive cues (Shalev, 2015).

According to MCI, this process occurs both in the bottom-up and the top-down directions through selective attention to multisensory information and affective signals, which, in turn, are integrated into meaning and result in action generation. Awareness of the association between high-level processes and low-level processes has been demonstrated in various studies. Based on the rationale that many voluntary actions aimed at ensuring homeostatic properties, Ainley and Tsakiris (2013) have shown that participants with higher levels of interoceptive awareness have demonstrated a greater intentional binding that reflects a stronger sense of agency. In addition, lower levels of interoceptive awareness were associated with greater self-other blurring during the enfacement illusion (Tajadura-Jiménez et al., 2012; Tajadura-Jiménez and Tsakiris, 2014). Additionally, Barrett et al. (2001) argued that persons with highly differentiated emotional experience could better regulate their emotions, pointing to the fact that a greater sensitivity to ongoing bodily changes will facilitate the regulation of emotional responses. This view is consistent with recent findings on the association between interoceptive ability and emotion regulation (Zamariola et al., 2019).

## Low-Level Cues and the Creation of Meaning

The distinction between high-level and low-level processes has been illustrated in earlier emotion theories (James and Dennis, 1884; Damasio, 1994; Craig, 2004). For example, psychological theory of James and Dennis (1884) associated visceral-afferent feedback and emotional experience. The assumption that higher level of processing is grounded in the organism’s lower level sensory and motor experiences is also at the core of embodied cognition research (Barsalou, 1999, 2008; Meier et al., 2012; Winkielman et al., 2015), enabling new ways to conceptualize and understand emotional processes (Damasio, 1999; Bechara and Damasio, 2005; Niedenthal, 2007). There is evidence that embodied cognition is influenced by various sources of information including innate processes, personal history, and culture (Meier et al., 2012). The general idea is that exeroceptive contextual cues activate associated mental representations (e.g., Bargh and Morsella, 2010; Loersch and Payne, 2011), suggesting that activation automatically spreads from concepts driven by experiences in the physical world to their metaphorically-related social concepts (for reviews, see Williams et al., 2009; Meier et al., 2012). For example, research of the emotion-related associations between physical warmth/coldness and psychological warmth/coldness was heterogeneous, indicating that momentary motivational states can lead to different patterns of activation across different situations (Bargh and Shalev, 2012; Shalev, 2015).

This variability in findings and the challenge of replication (Shalev and Bargh, 2015) help explain why MCI emphasized the relevance of both internal and external cues. Research on interoception and psychopathology indicates various associations between psychopathology and over-sensitivity vs. under-sensitivity to interoceptive cues (Khalsa et al., 2018). Accordingly, recent developments in neuroscience research indicate the existence of two types of inputs: (1) exteroceptive inputs associated with the perception of the body from the outside, based on multisensory integration and (2) interoceptive inputs, defined as the sense of the internal physiological state that supports homeostatic regulation of the body, resulting in physiological integrity and associated affective states, drives, and emotions (Garfinkel et al., 2015).

## High-Level Goals, Emotion, and Action Generation

MCI suggests that the monitoring of the flow of interoceptive and exeroceptive cues, associated with words, mental images, accessible memories along with the individual’s goals, will contribute to the formation of integrative meaning (Shalev, 2018). Goals are defined as hierarchically organized cognitive representations of desired endpoints that affect evaluations, emotions, and behaviors (Fishbach and Ferguson, 2007). Several alternative means to the same goal potentially exist and could substitute for each other (Kruglanski et al., 2002). The degree and length of situational activation of individual’s goals are influenced by sources of motivational relevance regardless of representation content. For example, value relevance is the extent to which acting on a mental representation produces desired results and/or prevents unwanted results; control relevance is related to the efficacy with which the activated representation makes things happen; and truth relevance determines what is real (Eitam and Higgins, 2010).

Individual differences are conveyed by the unique associations that individuals make between goals and means of attainment (Shalev, 2015), the repeated coupling of sensory signals (Rescorla, 1985), and the strength of the association between particular physical sensations and psychological concepts such as the combination of homeostatic cues (e.g., temperature and dryness). In addition, situational demands, history, and psychiatric and neuropsychological conditions (e.g., cognitive flexibility) influence cue integration (Shalev, 2015). Recent findings indicate, for example, that avoidance behavior in social anxiety may be related to biased distance estimation (Givon-Benjio and Okon-Singer, 2020).

There is evidence that both emotional and non-emotional action tendencies are determined by high-level goal-directed processes, which differ only in the degree of control precedence that they have (Hommel et al., 2017). Accordingly, competition between different goal-directed processes results in action control loops in which the degree to which a given stimulus event is related to and discrete from a current goal is assessed (Hommel et al., 2017; Moors et al., 2017). This helps to explain that emotional episodes are conceptualized as special types of goal-directed action episodes (Hommel et al., 2017). Similarly, Solms (2014) suggested that emotion is extended to exteroception (i.e., contextualized: “I feel like that”) and transformed into a goal which comes into play through voluntary action.

## Mci, Predictive Coding, and Low Emotional Clarity

According to the predictive coding model (e.g., Rao and Ballard, 1999; Clark, 2013; Spratling, 2017), emotional responses depend on the continuous updated process of prediction of internal signal causes. This process occurs because, to navigate the body in the world and minimize a free energy functional of internal states, the brain must discover information about the likely reasons for sensory cues (i.e., perception) without direct access to these causes (Friston, 2010). This process is also influenced by the need to minimize the cost of prediction error either by updating generative models or by performing actions to associate sensory states in line with predictions (Hutchinson and Barrett, 2019). For example, upon detecting physical or cognitive change, probabilistic inference on the reasons for sensory cues is computed according to Bayesian principles such that the brain may represent the concept of “anger” as having a 90% chance of accounting for that pattern, while representing “sadness” as having a 5% chance, happiness a 1% chance, and so forth (Barrett, 2017; Smith et al., 2017). From the perspective of interoceptive predictive coding models (Seth, 2013), interoceptive prediction error arises when the “actual” state of the body does not match the predicted state.

MCI suggests that dissociation between high-level personal goals and low-level multisensory data results in a prediction error. Under this condition, automatic emotion regulation of preferences does not contribute to relief of psychological distress. While automatic prediction may be efficient in different contexts, low emotional clarity requires increased awareness.

Based on this reasoning, according to MCI, low access to multisensory data could be viewed as a means substitution problem (Kruglanski et al., 2002), indicating that when goal progress *via* a prior means was unrecognized or thwarted, alternative means should be used. Following this view, alternative types of encoding (e.g., mental images and multisensory data) may increase access to the dissociated aspects of the psychological experience and clarify emotion. Supportive evidence exists for the possible substitutability of emotion and alternative types of encoding, indicating the association between multisensory data and mental images as alternative types of encoding (Holmes and Mathews, 2005). For example, research shows that mental images in different modalities are primarily sensory-perceptual representations. There is overlap between the brain areas activated during imagery and those involved in processing the equivalent sensory and perceptual events. For example, there is evidence that perceptual cues associated with homeostatic deficiency (e.g., words and visual images of dryness) influence self-regulation as if they were actual ones (Shalev, 2014, 2016; Halali et al., 2017). Likewise, neural processes involved in perceiving “real” events (Kosslyn et al., 2001) signaling danger or reward (Öhman and Mineka, 2001) and mental imagery areas are shared.

## Mci and the Creation of Emotion Goal

To increase clarity of emotion, a moment-to-moment self-regulation should be based on tracking accessible multisensory data and creation of association between selective perceptual cues and individual need, suggesting that low emotional clarity results in self-regulation failure. While in predetermined situations, individuals are governed by automatic reflexive responses, if the person wants to make plausible decisions in unfamiliar circumstances, this will be achieved through a sort of integration of past experience and current alternatives in affective evaluation (Solms, 2014). Accordingly, emotional clarity enables complex organisms to identify, monitor, and handle deviations from homeostatic settlement points in uncertain contexts through integrative comprehension and voluntary action. Such change underpins learning from experience (Solms and Friston, 2018).

Although most emotion-regulating approaches address individual maladaptive feelings, MCI proposes treating emotion control as a function of contextual meaning. The general idea is that low emotional clarity caused low access to multisensory data. Therefore, awareness of sensation techniques may support the re-creation of meaning. To address this goal, the individual should first identify a situation or event that caused discomfort and invest selective attention in the physical sensations, associations, and mental images associated with this event. Techniques involve awareness-of-sensation to peripheral cues (e.g., mindfulness and experiential approach), enabling emergence of new details which may reconstruct the emotional experience. Gendlin (2012) suggested that a positive shift in psychological experience emerges from tracking the changes of bodily experience. As such, in the practice of focusing, the focuser assigns words, mental images, or phrases that express the present sensory experience. The focuser ranges from feeling to verbal association and returning to other physical sensations that arise during the process. After remaining throughout the interaction, a shift is generated and a greater understanding of the essence of the problem or action should be taken. Attention to perceptual cues results in the identification of a specific word or image that carries meaning or reveals an individual’s unconscious personal purpose. Once relief has been achieved, the focuser labels items that she wants to memorize from the process or to formulate an integrative image of the experience.

Clinicians and neuropsychologists can train individuals to use awareness-of-sensation techniques to increase emotional clarity by integrating peripheral multisensory data and creating associations between perceptual cues, symbols, and the individual’s personal needs to create an emotion regulation goal. Integrating peripheral cues in the moment-by-moment creation of meaning may bridge the gap between research on higher level emotion regulation (Suri et al., 2018) and various conditions that hinder personal goal accessibility. There is evidence that use of brief awareness of sensation enhances aspects of emotional processing such as emotional intensity, emotional memory, and emotional attention bias (Guendelman et al., 2017; Wu et al., 2019). Research indicates that mindfulness influences interpersonal emotional reactions through an experiential process, while altering the subjective and physiological experience of emotions, and also biasing interpersonal behavioral patterns (Grecucci et al., 2015). Likewise, there is evidence that when expecting negative images, prefrontal and right insular activations correlated negatively with trait-mindfulness, indicating that more attentive individuals need fewer regulatory resources to attenuate emotional arousal (Lutz et al., 2014). Future research should further study the relations between higher level individual goals and lower level interoceptive and exteroceptive signals to increase emotional clarity and to shed light on individual’s needs. Integrating high-level processes and low-level signals may diagnose various psychological conditions and add to the state-of-the-art self-regulation research.

## Author Contributions

The author confirms being the sole contributor of this work and has approved it for publication.

## Conflict of Interest

The author declares that the research was conducted in the absence of any commercial or financial relationships that could be construed as a potential conflict of interest.
